# Supplementary figures and images for: Prevalence and risk predictors of childhood stunting in Bangladesh
Source: PLoS One. 2023 Jan 26;18(1):e0279901. doi: 10.1371/journal.pone.0279901 (PMC9879476; doi:10.1371/journal.pone.0279901)

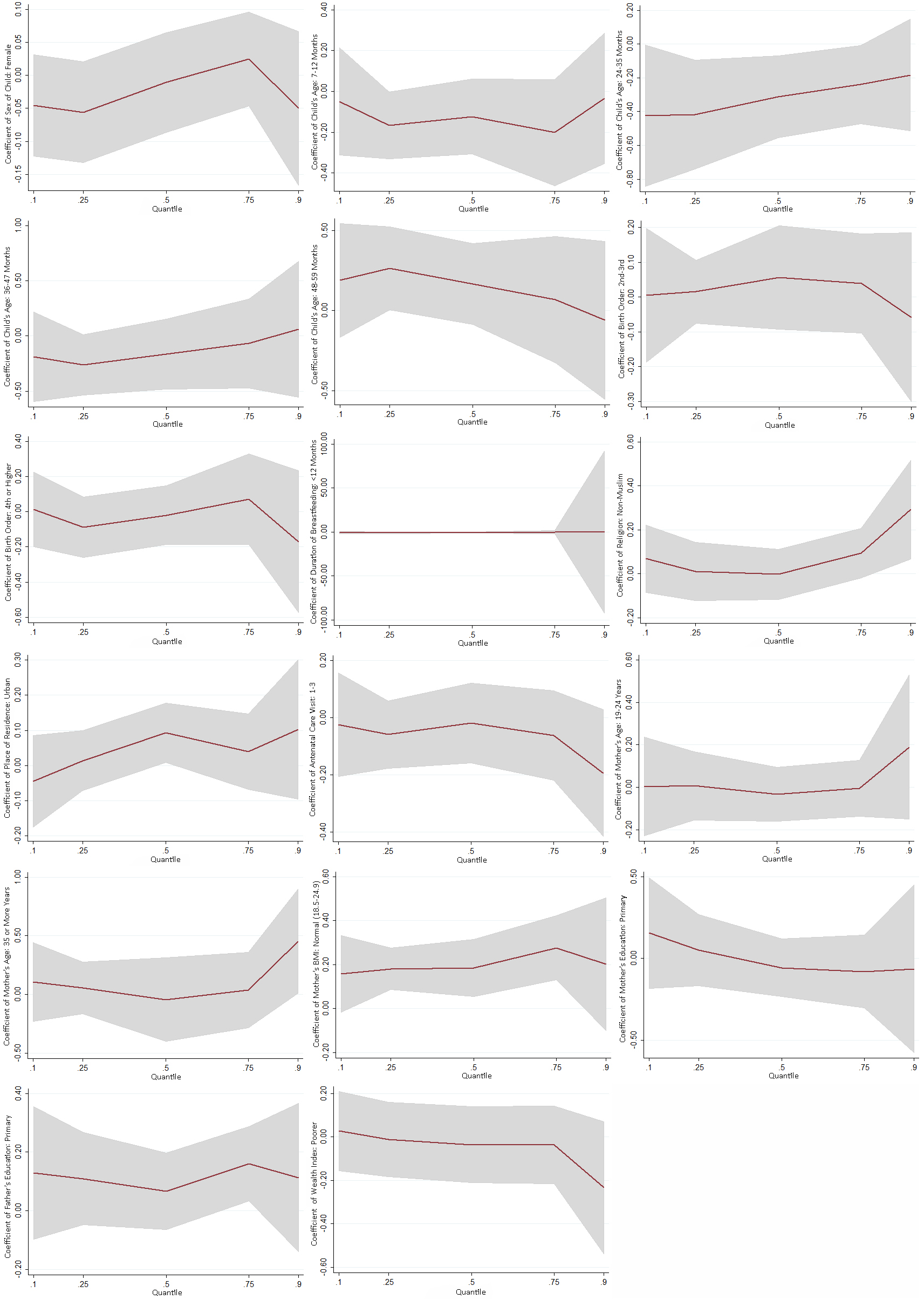

Supplement: S1 Fig — (TIF) [file pone.0279901.s001.tif]
