# Supplementary material for: Prevalence and risk predictors of childhood stunting in Bangladesh
Source: PLoS One. 2023 Jan 26;18(1):e0279901. doi: 10.1371/journal.pone.0279901 (PMC9879476; doi:10.1371/journal.pone.0279901)
Supplement: Attachment — Submitted filename: PONE-D-22-12168_reviewed.pdf [file pone.0279901.s003.pdf]

PLOS ONE

Prevalence and Risk Predictors of Childhood Stunting in Bangladesh

--Manuscript Draft--

**Manuscript Number:**	PONE-D-22-12168
**Article Type:**	Research Article
**Full Title:**	Prevalence and Risk Predictors of Childhood Stunting in Bangladesh
**Short Title:**	Prevalence and Risk Predictors of Childhood Stunting
**Corresponding Author:**	Md. Moyazzem Hossain<br>Jahangirnagar University<br>Savar, Dhaka, BANGLADESH
**Keywords:**	Anthropometric indicators; Bangladesh; Quantile regression; Socioeconomic factors; Sustainable development goals
**Abstract:**	The child nutritional status of a country is performing as a potential indicator of the socioeconomic development. This paper investigates the relationship between demographic and socio-economic factors and nutritional status of under-5 children in Bangladesh and differentiates significant risk predictors of stunting with a quantile regression approach. The study performs the analysis based on secondary data of the BDHS-2017-18. The height-for-age (HAZ) z-score based anthropometric indicator was used in the study as the target variable. The BDHS-2017-18 sample was collected with two stages stratified sampling technique. Firstly, the enumeration areas (EAs) were chosen with probability proportional to the EA size and finally, 30 households per cluster were chosen systematically with an equal probability. The sample represents 20,127 ever-married women aged 15-49 years and 8,334 children under-5 years. However, after cleaning the missing values, the analysis is based on 8,321 children under-5 years of age. The findings of quantile regression demonstrated that birth order was significantly and inversely associated with the HAZ score. The mother's age and BMI, father's education and household's wealth index were positively associated with the HAZ score. However, the extent of these associations also considerably varied throughout the conditional distribution of the HAZ score. To enhance the nutritional level of children who lived in Bangladesh, the contributors of this paper recommend a mutual effort by the government, NGOs and different communities to improve the identified risk determinants for child malnutrition. Improving these significant determinants would accelerate the achievement of targets set by the Sustainable Development Goals (SDGs) related to child and maternal health in Bangladesh by 2030.
**Order of Authors:**	Faruq Abdulla
	Azizur Rahman
	Md. Moyazzem Hossain
**Additional Information:**	
**Question**	**Response**
**Financial Disclosure**<br><br>Enter a financial disclosure statement that describes the sources of funding for the work included in this submission. Review the submission guidelines for detailed requirements. View published research articles from *PLOS ONE* for specific examples.<br><br>This statement is required for submission and **will appear in the published article** if	The author(s) received no specific funding for this work.

Powered by Editorial Manager® and ProduXion Manager® from Aries Systems Corporation

the submission is accepted. Please make sure it is accurate.<br><br>**Unfunded studies**<br>Enter: *The author(s) received no specific funding for this work.*<br><br>**Funded studies**<br>Enter a statement with the following details:<br>• Initials of the authors who received each award<br>• Grant numbers awarded to each author<br>• The full name of each funder<br>• URL of each funder website<br>• Did the sponsors or funders play any role in the study design, data collection and analysis, decision to publish, or preparation of the manuscript?<br>• **NO** - Include this sentence at the end of your statement: *The funders had no role in study design, data collection and analysis, decision to publish, or preparation of the manuscript.*<br>• **YES** - Specify the role(s) played.<br><br>* typeset	
**Competing Interests**<br><br>Use the instructions below to enter a competing interest statement for this submission. On behalf of all authors, disclose any competing interests that could be perceived to bias this work—acknowledging all financial support and any other relevant financial or non-financial competing interests.<br><br>This statement is required for submission and **will appear in the published article** if the submission is accepted. Please make sure it is accurate and that any funding sources listed in your Funding Information later in the submission form are also declared in your Financial Disclosure statement.<br><br>View published research articles from *PLOS ONE* for specific examples.	The authors have declared that no competing interests exist.

**NO authors have competing interests**<br><br>Enter: *The authors have declared that no competing interests exist.*<br><br>**Authors with competing interests**<br><br>Enter competing interest details beginning with this statement:<br><br>*I have read the journal's policy and the authors of this manuscript have the following competing interests: [insert competing interests here]*<br><br>* typeset	
**Ethics Statement**<br><br>Enter an ethics statement for this submission. This statement is required if the study involved:<br><br>• Human participants<br>• Human specimens or tissue<br>• Vertebrate animals or cephalopods<br>• Vertebrate embryos or tissues<br>• Field research<br><br>Write "N/A" if the submission does not require an ethics statement.<br><br>General guidance is provided below. Consult the submission guidelines for detailed instructions. **Make sure that all information entered here is included in the Methods section of the manuscript.**	N/A

**Format for specific study types**<br><br>**Human Subject Research (involving human participants and/or tissue)**<br>• Give the name of the institutional review board or ethics committee that approved the study<br>• Include the approval number and/or a statement indicating approval of this research<br>• Indicate the form of consent obtained (written/oral) or the reason that consent was not obtained (e.g. the data were analyzed anonymously)<br><br>**Animal Research (involving vertebrate animals, embryos or tissues)**<br>• Provide the name of the Institutional Animal Care and Use Committee (IACUC) or other relevant ethics board that reviewed the study protocol, and indicate whether they approved this research or granted a formal waiver of ethical approval<br>• Include an approval number if one was obtained<br>• If the study involved *non-human primates*, add *additional details* about animal welfare and steps taken to ameliorate suffering<br>• If anesthesia, euthanasia, or any kind of animal sacrifice is part of the study, include briefly which substances and/or methods were applied<br><br>**Field Research**<br><br>Include the following details if this study involves the collection of plant, animal, or other materials from a natural setting:<br>• Field permit number<br>• Name of the institution or relevant body that granted permission	
**Data Availability**<br><br>Authors are required to make all data underlying the findings described fully available, without restriction, and from the time of publication. PLOS allows rare exceptions to address legal and ethical concerns. See the PLOS Data Policy and FAQ for detailed information.	Yes - all data are fully available without restriction

A Data Availability Statement describing where the data can be found is required at submission. Your answers to this question constitute the Data Availability Statement and **will be published in the article**, if accepted.<br><br>**Important:** Stating 'data available on request from the author' is not sufficient. If your data are only available upon request, select 'No' for the first question and explain your exceptional situation in the text box.<br><br>Do the authors confirm that all data underlying the findings described in their manuscript are fully available without restriction?	
**Describe where the data may be found in full sentences. If you are copying our sample text, replace any instances of XXX with the appropriate details.**<br><br>• If the data are **held or will be held in a public repository**, include URLs, accession numbers or DOIs. If this information will only be available after acceptance, indicate this by ticking the box below. For example: *All XXX files are available from the XXX database (accession number(s) XXX, XXX.*).<br>• If the data are all contained **within the manuscript and/or Supporting Information files**, enter the following: *All relevant data are within the manuscript and its Supporting Information files.*<br>• If neither of these applies but you are able to provide **details of access elsewhere**, with or without limitations, please do so. For example:<br><br>*Data cannot be shared publicly because of [XXX]. Data are available from the XXX Institutional Data Access / Ethics Committee (contact via XXX) for researchers who meet the criteria for access to confidential data.*<br><br>*The data underlying the results presented in the study are available from (include the name of the third party*	This study is based on the secondary dataset. One can access the data set via the following link http://dhsprogram.com/data/available-datasets.cfm.

and contact information or URL).<br>• This text is appropriate if the data are owned by a third party and authors do not have permission to share the data.<br><br>* typeset	
Additional data availability information:	

Manuscript Click here to access/download;Manuscript;Manuscript_22.04.2022.docx

Prevalence and Risk Predictors of Childhood Stunting in Bangladesh

Faruq Abdulla[1], Azizur Rahman[2] and Md. Moyazzem Hossain[3,4*]

[1]Department of Applied Health and Nutrition, RTM Al-Kabir Technical University, Sylhet, Bangladesh; E-mail: faruqiustat09mnil@gmail.com

[2]School of Computing, Mathematics and Engineering, Charles Sturt University, Wagga Wagga, NSW 2678, Australia; Email: azrahman@csu.edu.au

[3]Department of Statistics, Jahangirnagar University, Savar, Dhaka-1342, Bangladesh; Email: hossainmm@juniv.edu

[4]School of Mathematics, Statistics and Physics, Newcastle University, Newcastle upon Tyne, UK

*Corresponding author

Abstract

The child nutritional status of a country is performing as a potential indicator of the socioeconomic development. This paper investigates the relationship between demographic and socio-economic factors and nutritional status of under-5 children in Bangladesh and differentiates significant risk predictors of stunting with a quantile regression approach. The study performs the analysis based on secondary data of the BDHS-2017-18. The height-for-age (HAZ) z-score based anthropometric indicator was used in the study as the target variable. The BDHS-2017-18 sample was collected with two stages stratified sampling technique. Firstly, the enumeration areas (EAs) were chosen with probability proportional to the EA size and finally, 30 households per cluster were chosen systematically with an equal probability. The sample represents 20,127 ever-married women aged 15-49 years and 8,334 children under-5 years. However, after cleaning the missing values, the analysis is based on 8,321 children under-5 years of age. The findings of quantile regression demonstrated that birth order was significantly and inversely associated with the HAZ score. The mother's age and BMI, father's education and household's wealth index were positively associated with the HAZ score. However, the extent of these associations also considerably varied throughout the conditional distribution of the HAZ score. To enhance the

nutritional level of children who lived in Bangladesh, the contributors of this paper recommend a mutual effort by the government, NGOs and different communities to improve the identified risk determinants for child malnutrition. Improving these significant determinants would accelerate the achievement of targets set by the Sustainable Development Goals (SDGs) related to child and maternal health in Bangladesh by 2030.

Keywords: Anthropometric indicators; Bangladesh; Quantile regression; Socioeconomic factors; Sustainable development goals

1. Background

Nutritional status is the result of aggregate connections between consumption of food, care practices and overall health conditions. Sufficient nutrition is the precondition for accomplishing sound health, quality of life, as well as national productivity. Even though complications arise due to poor nutrition affect the whole population, women as well as children have been exposed especially for the reason of their socio-economic and unique physiological characteristics. The consequences of insufficient nutrition, however, are forwarded from generation to generation, as undernourished girls have a tendency to become shorter adults and consequently are more probable to have small offspring [1]. Stunting has both short as well as long term impacts on children in direct and indirect ways, such as low birth weight, obstructing cognitive development which affect school achievement, and restricting the prospects of their life in adulthood [2]. Stunting can also affect a child's social personal development [3].

The child nutritional status of a country is performing as a potential indicator of the socioeconomic development. However, a low nutritional level is the main cause of critical health as well as welfare problems facing in Bangladesh and trying very hard to lessen child malnutrition and managed to achieve some success. The BDHS reports indicate that the rate of stunting child is declined by 12 percentage points between 2007 and 2017-18 [4]. Stunting (i.e. a low linear growth) is considered to be a considerable public health problem among children in many countries [5]. Globally, about 150.8 million of children aged under-5 years are stunted in 2017

[6], and nearly about 40 percent of stunted children lived in Southern Asia [7]. The serious consequences of stunting have led to the establishing of worldwide nutrition targets to lessen the prevalence of stunted children under-5 years of age by 40% before 2025 [8]. This worldwide target has since been subsequently supported by the Sustainable Development Goal 2 (SDG-2), target 2 and committed to end all kinds of malnutrition by the year 2030 and avoiding at least 1.7 million childhood deaths with the help of taking different prevention of stunting [9,10]. Furthermore, before COVID-19, it is mentioned that the current world was already off-track to accomplish the Sustainable Development Goal 2 to reduce hunger as well as all types of malnutrition by the year 2030, however, because of COVID-19 pandemic, the economic recession will faced worldwide which will be impacted hugely to meet up the global nutritional targets for stunting by 2025 specially in low and middle income countries [11,12]. About 2.6 million of children additionally will be stunted in 2022 compared due to disruptions in nutrition services as well as drops in household poverty status [13]. Fawzi et al. (2019) mention that about 7.2 million of stunting child who lived in low/middle-income countries were due to psychosocial factors [14]. Many previous studies showed that stunting risk is associated with sex, age, low birth weight, children suffering from diarrhea, religious status, geographical locations, maternal or parental education, maternal or parental stature, wealth index, mother's BMI, breastfeeding status, child's immunization status, food security and eating behaviours, mycotoxins, family size, household environment and facilities, Gross Domestic Product, intimate partner violence and media exposure [2,6,22–31,14,32–41,15,42–50,16–21]. Moreover, several studies are available related to child malnourishment in Bangladesh [51–55].

However, most of these previous studies have applied either binary regression or linear regression models to explore the potential factors of child malnutrition. However, the average influence may over or underestimate the effect of the selected covariates at different points. Additional critical limitation of the binary regression model is that it treats observations equally that are above or below a threshold point and overlooks the intensity of the variations from that threshold point. As a consequence, there may possibly be a loss of information which may be relevant to intervention as well as the measures of health promotion. Though, the quantile regression (QR) model provides the robust estimates in the presence of outliers, and it not only worry with the mean as well as

median behaviour [56]. Besides, the QR model produced estimates that are relatively more unbiased than the estimates generated by the linear regression model when the data violate the assumption of normality [57]. Hossain & Majumder (2019) applied the quantile regression for assessing the potential correlates of the age of the mother at first birth because it is not normally distributed [58]. Also, Borooah (2005) applied QR regression for capturing the heterogeneity as well as the determinants of height-for-age in India and pointed out that access to safe water and improved hospital facilities enhance z-scores of anthropometric measurements of a child at the smallest quantiles [59]. Furthermore, Aturupane et al. (2011) analyse the determining factor of a child's height and weight with the help of quantile regression in Sri Lanka [60]. Fenske et al. (2013) carry out a comprehensive assessment of the underlying factors of child stunting status in India by the QR model [61]. Sharaf et al. (2019) also examine the underlying demographic as well as socio-economic factors of the nutritional status of the child in Egypt by QR model [62].

Moreover, in this study, the coefficient of skewness of the target variable depicts that the HAZ score does not hold the normality assumption. This is the reason working behind to select the QR model for analyzing the data. Therefore, this paper attempt to investigate the correlation between demographic as well as socio-economic factors and the nutritious status of children having age less than 5 years in Bangladesh using Quantile Regression Models considering the secondary data collected from the latest BDHS-2017-18. The existing literatures indicate that a limited research is available on this vital topic with Bangladeshi data considering QR model. The Z-score of height-for-age is employed to measure child growth or stunting, and the quantile regression analysis is applied to determine the effect of demographic as well as socio-economic variables of the study children. The findings of this research will be helpful in designing the effective intervention measures targeted at preventing child malnutrition and enhancing child health status which will also be supportive of accelerating the achievement of targets set by the Sustainable Development Goals (SDGs) linked to child health status in Bangladesh by the year 2030.

2. Methods

2.1 Data and Variables

In this study the secondary data is obtained from a nationally representative survey called the 2017-18 Bangladesh Demographic and Health Survey (BDHS-2017-18). The BDHS-2017-18 is the complete survey that covers the enumeration areas (EAs) of the whole country. The BDHS-

2017-18 used stratified sampling and selection is made in two stages. Firstly, 675 EAs were chosen with probability proportional to the EA size. In the second phase of selection, 30 households per cluster were carefully chosen with an equal probability-based systematic procedure from the list of households. However, data was not possible to collect from 3 EAs due to natural disaster. These three clusters were in Rajshahi (one rural cluster), Rangpur (one rural cluster), and Dhaka (one urban cluster). The sampling procedure is available on the published reports in details [4].

The child nutritional status in the survey population has compared with the Child Growth Standards recommended by the World Health Organization (WHO), which is constructed on an international sample of culturally, ethnically, and genetically distinct healthy children residing under optimum environments that are favorable to achieve a child's full genetic growth potential [4]. Among the three main anthropometric indexes for child growth, height-for-age measures the linear growth. A child is likely to be a stunted child if s/he has a height more than two standard deviations below the reference median of height for that age. As with stunting is considered severe if the child is more than three standard deviations below the reference median of height for that age [4]. The height-for-age Z-score (HAZ) is the target variable, and several child characteristics such as sex, age, duration of breastfeeding, birth order,; maternal attributes such as age, education and BMI; father's education, and attributes related to household, community along with health are the explanatory variables in this study. The selection of variables used in this study was motivated by the availability in the BDHS dataset and self-efficacy as well as guided by relevant literature.

2.2 Quantile Regression

The quantile regression (QR) model was initially introduced by Koenker and Basset in 1978, and nowadays it is extensively applied in various research areas, particularly in Statistics and Econometrics [63]. Suppose, Y be a random (response) variable having cumulative distribution function (CDF) $F_Y\left(y\right)$, i.e. $F_Y\left(y\right)=P\left(Y\le y\right)$ and X is the p-dimensional vector of predictor variables. Then the τ th conditional quantile of Y is described as

$$Q_\tau\left(Y \middle| X = x\right) = \left\{y : F_\tau\left(y \middle| x\right)\right\},$$

where the quantile level τ varies from 0 to 1.

The QR model portrayed by the conditional τ th quantiles of the response Y for considering the values of predictors $x_1, x_2, ..., x_p$ can be expressed as $Q_y\left(\tau \middle| x_1, x_2, ..., x_p\right) = \beta_0^{(\tau)} + \beta_1^{(\tau)} x_1 + ... + \beta_p^{(\tau)} x_p, 0 < \tau < 1$,

where $\beta^{(\tau)} = \left(\beta_0^{(\tau)}, \beta_1^{(\tau)}, ..., \beta_k^{(\tau)}\right)^T$ is the unknown vector of parameters.

For a random sample $\{y_1, y_2, ..., y_n\}$ of Y, it is understood that the sample median minimises the following sum of absolute deviations, $Median = \arg\min_{\xi\in\mathbb{R}} \sum_{i=1}^{n} \rho_\tau\left(y_t - \xi\right)$. Likewise, the general τ th sample quantile $\xi(\tau)$, which is the analogue of $Q(\tau)$, is formulated as the minimiser: $\xi(\tau) = \arg\min_{\xi\in\mathbb{R}} \sum_{i=1}^{n} \rho_\tau\left(y_t - \xi\right)$, where $\rho_\tau(Z) = Z\left(\tau - I\left(Z < 0\right)\right)$ for $0 < \tau < 1$ denotes the loss function with an indicator function $I(.)$. The loss function ρ_τ allocates a weight of τ and $1-\tau$ for positive residuals = $y_i - \xi$ and negative residuals respectively. The linear conditional quantile function along with this loss function expands the τ th sample quantile $\xi(\tau)$ to the regression setting in the similar way that the linear conditional mean function expands the sample mean. The OLS estimates is obtained based on the linear conditional mean function $E\left(Y \middle| X = x\right) = x'\beta$, by solving $\hat{\beta} = \arg\min_{\beta\in\mathbb{R}^p} \sum_{i=1}^{n} \left(y_t - x'\beta_i\right)^2$ [64].

The estimated parameter minimises the sum of squared residuals as the sample mean minimises the sum of squares $\mu = \arg\min_{\mu\in\mathbb{R}} \sum_{i=1}^{n} \left(y_t - \mu\right)^2$. Quantile regression also estimates the linear conditional quantile function, $\left(\tau \middle| X = x\right) = x'\beta(\tau)$, by solving $\hat{\beta}(\tau) = \arg\min_{\beta\in\mathbb{R}^p} \sum_{i=1}^{n} \rho_\tau\left(y_t - x'\beta_i\right)^2$. For any quantile $\tau \in (0,1)$ the quantity $\hat{\beta}(\tau)$ is known as

the τ th regression quantile. For example $\tau = 0.5$, which minimises the sum of absolute residuals, and also corresponds to L_1-type or median regression. The set of regression quantiles $\{\beta(\tau) : \tau \in (0,1)\}$ is called the quantile process [64].

The QR model aimed at solving the term $\min_{\beta \in \mathbb{R}^p} \left[\sum_i \tau |e_i| + \sum_i (1-\tau)|e_i| \right]$, where $e_i = y_i - x_i'\beta$ is the i th value of unknown errors, $\sum_i \tau |e_i|$ gives the asymmetric penalties $\tau |e_i|$ for over prediction and $\sum_i (1-\tau)|e_i|$ gives the asymmetric penalties $(1-\tau)|e_i|$ for under prediction [64]. The τ th quantile regression estimator $\hat{\beta}(\tau)$ is obtained by minimising the following objective function over β_τ

$$Q(\beta_\tau) = \sum_{i \in \{i: y_i \geq x_i'\beta\}}^{N} \tau |y_i - x_i'\beta_\tau| + \sum_{i \in \{i: y_i < x_i'\beta\}}^{N} (1-\tau)|y_i - x_i'\beta_\tau|$$

where, $0 < \tau < 1$, $i : y_i \geq x_i'\beta$ for over prediction, $i : y_i < x_i'\beta$ for under prediction [64].

3. Results

This study considered the Z-score of height-for-age as the target variable. Figure 1 depicts the height-for-age (HA) Z-scores against the standard normal variate. Results revealed that the average and standard deviation of HAZ score are -1.206 and 1.22, respectively, with the coefficient of skewness and kurtosis are 0.25 and 1.05, respectively. The average of the HAZ-score is less than 0 recommends that the distribution of HAZ index has moved downward and the majority of the children are suffering from stunting malnutrition relative to the reference population. The coefficient of skewness of the HAZ-scores demonstrates that the distribution of the HAZ-score is positively skewed for the under-5 children in Bangladesh.

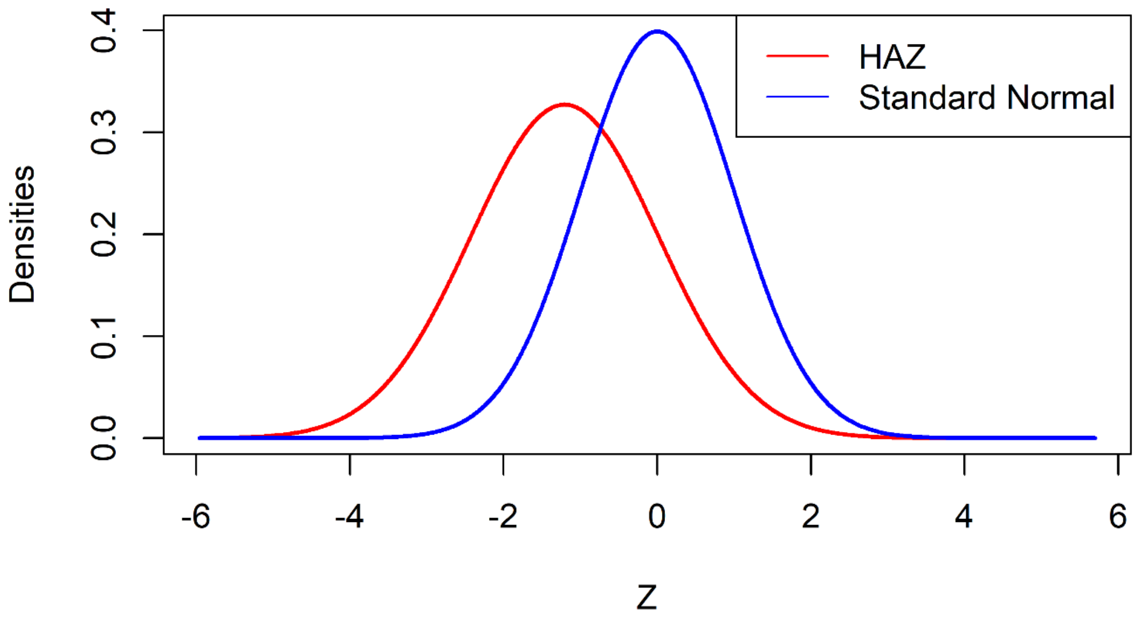

Figure 1: Density plot of the height-for-age (HAZ) Z-scores and the standard Normal variate.

Table 1 presents the prevalence of stunted child among Bangladeshi children aged under-5 years along with different characteristics considered in this study. The findings depict that overall about 18% of children were stunted (i.e. HAZ-score < -2 SD), and approximately 6% of children were severely stunted (i.e. HAZ-score < -3 SD) in Bangladesh. In the child characteristics, about 52.2% of children are male, and their prevalence rate of severe stunting is relatively less than female children. More than 13% of children have aged less than or equal to 6 months, and the rate of stunting typically increased with the child's age up to two years, however, after the second birthday of the child the rate of severe stunting decreases slightly. More than one-third of children having age less than 5 years in Bangladesh were at the first birth order, and this variable exhibited a positive association with the childhood stunting. Muslim child is slightly more stunted than their counterpart. Almost half of children are still breastfeeding (see Table 1). The findings disclose that the breastfeeding duration is negatively associated with the percentage of severely stunted child (i.e. HAZ-score < -3 SD).

Table 1: Prevalence of child categorized according to the anthropometric index height-for-age (stunting) by selected characteristics, Bangladesh (BDHS 2017-18, n = 8,321).

Background characteristics		Percent	Height-for-Age (Stunting) in %		
			Severe Stunted (Z<-3 SD)	Moderately Stunted (Z<-2 SD)	P value
Child characteristics					
Sex	Male	52.2	5.26	17.95	0.086
	Female	47.8	6.68	18.24	
Child's age (in months)	<= 6	13.14	0.90	8.00	<0.001
	7-12	9.94	3.20	13.80	
	13-23	18.45	8.30	24.10	
	24- 35	19.87	6.80	18.80	
	36-47	19.16	6.60	19.80	
	48-59	19.44	7.10	19.20	
Birth order	1st	38.3	4.90	17.70	<0.001
	2nd-3rd	49.2	5.70	17.10	
	4th and higher	12.5	10.00	23.30	
Duration of breastfeeding	Never breastfeed	41.30	7.30	19.40	<0.001
	<12 months	2.10	4.80	18.80	
	12 or more	7.00	4.80	19.20	
	Still breastfeeding	49.7	5.10	16.80	
Religion	Muslim	92.00	6.00	18.10	0.091
	Non-muslim	8.00	5.50	17.80	
Parental Characteristics					
Mother's age (Years)	Up to 18	7.20	4.50	18.50	<0.001
	19-24	40.20	5.20	18.70	
	25-34	44.70	6.70	17.40	
	35+	7.90	6.90	18.50	
Mother's BMI	Underweight (<18.5)	13.60	8.70	24.80	<0.001
	Normal (18.5-24.9)	59.22	6.00	18.70	
	Overweight (>=25)	27.18	4.40	13.10	
Mother's Education level	No Education	7.15	12.60	23.90	<0.001
	Primary	28.40	7.90	23.00	
	Secondary and above	64.45	4.30	15.20	
Father's Education level	No Education	14.85	10.90	24.80	<0.001
	Primary	34.29	7.20	21.20	
	Secondary and above	50.86	3.60	13.90	
Household and health characteristics					
Place of residence	Rural	73.04	6.20	19.60	<0.001
	Urban	26.96	5.10	14.00	
Place of delivery	With Health Facility	49.91	3.70	14.30	<0.001
	Respondent's Home	50.09	7.00	20.10	
Number of Antenatal visits during pregnancy	No antenatal visits	13.13	6.50	17.10	0.001
	1-3	44.66	5.40	18.80	
	4-7	36.18	4.30	15.70	

Background characteristics		Percent	Height-for-Age (Stunting) in %		
			Severe Stunted (Z<-3 SD)	Moderately Stunted (Z<-2 SD)	P value
	7+	6.03	2.90	13.00	
Had diarrhea recently	No	95.26	5.90	18.20	0.107
	Yes	4.74	6.90	16.60	
Had fever in last two weeks	No	66.79	5.60	18.20	0.325
	Yes	33.21	6.60	18.00	
Had cough in last two weeks	No	64.01	5.90	18.30	0.274
	Yes	35.99	6.00	17.70	
Received BCG	No	6.92	1.50	8.00	<0.001
	Yes	93.08	5.70	17.90	
Received Vitamin A	No	30.04	2.90	13.20	<0.001
	Yes	69.96	6.50	18.90	
Wealth index	Poorest	21.44	8.50	24.30	<0.001
	Poorer	20.33	7.60	21.80	
	Middle	18.86	4.70	18.70	
	Richer	19.88	5.80	15.10	
	Richest	19.48	2.50	9.20	
**Total (Overall)**			5.9	18.1	

Within the parental characteristics, more than 7% of children mothers were young (i.e. up to 18 years old), 13.6% of children mothers were underweight and more than a half of the children mothers have had a minimum of secondary level of education. Results also reveal that while the age of mother showed a positive relationship with the occurrence of stunting, both the nutritional status (i.e. BMI) and educational status of mother and father demonstrated a negative association with the stunting rate. Furthermore, concerning the household and health characteristics, results reveal that about three-fourth of the children who lived in rural areas and a higher incidence of stunting has observed in that region compared to urban regions. Place of delivery is also a significant factor for the nutritional level of children. Slightly half (i.e. 49.91%) of children have had a birth with the support of health facilities which have shown a lower stunting rate than who born at home. Number of antenatal care visits during pregnancy is also a key factor of stunting as the number of visit increases the prevalence of stunting is decreases. A lower percentage of study children had suffered from common diseases such as diarrhea (4.74%), fever (33.21%) and cough (35.99%). Figures also demonstrate that nutritional status of a child was related to the present health condition since the child who had fever and suffered from diarrhea has slightly more chance of stunting than the child without suffered from those diseases though the results depict the association is insignificant. Besides, about 41.6% of children were living in a low wealth

index household, and the percentage of stunting is inversely associated with increasing wealth index of household in Bangladesh [Table 1].

The study has considered the quantile of 0.10, 0.25, 0.50, 0.75 and 0.90 based on the previous literature [65]. Results of the quantile regression estimation for HAZ or stunting of under-5 children in Bangladesh are provided in Table 2. Data reveal significant differentials in the impacts of chosen study variables at various points of the conditional distribution of HAZ-score. In particular, sex of a child depict a significant effects on HAZ accept at the 50th quantile. Age of child is significant for all quantiles with age effects are lower at 0.10^{th} quantile compare to that at 0.90^{th} quantile, except for the age of 48-59 months. As we forward from the age of a child up to six months to age of 36 to 47 months, HAZ value has decreased by 0.188 points at 10th quantile, 0.166 point at 50th quantile whereas increased by 0.062 points at 90th quantile. When we compared the age of 24-35 months and age up to six months, HAZ is decreased by 0.423 and 0.184 points at the 10th and 90th percentile respectively.

Moreover, by comparing the breastfeeding status of a child, like never breastfed child with the child whose breastfeeding duration is more than one year, it is seen that there is a negative effect of breastfeeding at lower quantile. Several maternal attributes like mother's age, education level and BMI have significant and highly positive impacts on HAZ–score of a child at most of the quantiles considered in this study. The increase of the mother's age improves the HAZ value. As we go from underweight mothers to mothers who has normal BMI, the HAZ score raises 0.158 and 0.276 points at 10th and 75th quantile respectively. Interestingly, the current location of residence is adversely linked to HAZ value at lower quantile, however, it is positively associated with upper quantiles. Besides, place of delivery, the status of receiving BCG, vitamin A and number of visit antenatal care in pregnancy period show a significant association to the HAZ–score of a child. The results depict that household income is an essential factors of child health status and has a positive contribution in most of the quantile [Table 2]. Moreover, the extent of this association also differed across the conditional distribution of HAZ score (refer to Figure 2 and Supplement Figure 1) for determining childhood malnutrition in Bangladesh.

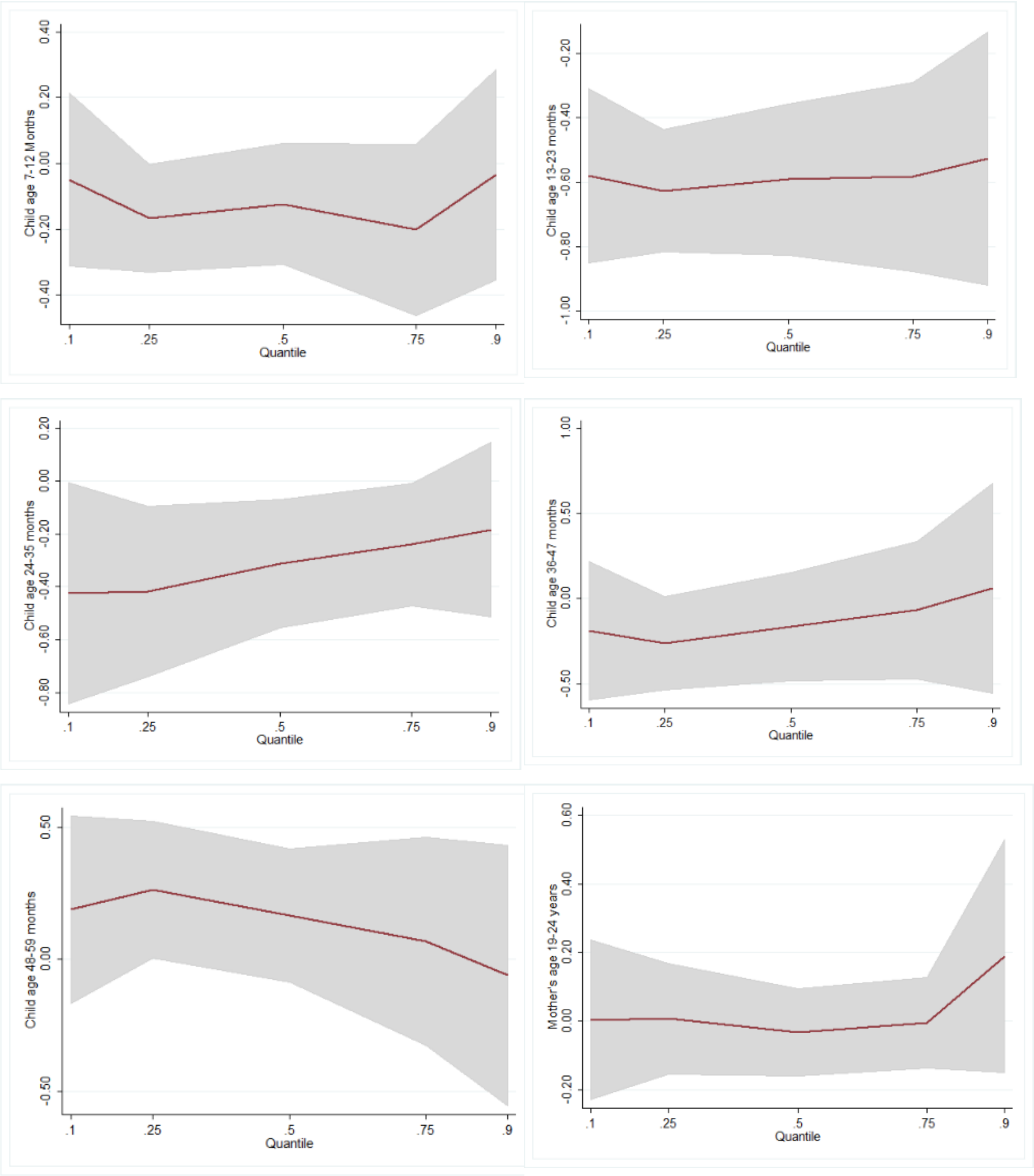
Child age 7-12 Months
Quantile
Child age 13-23 months
Quantile
Child age 24-35 months
Quantile
Child age 36-47 months
Quantile
Child age 48-59 months
Quantile
Mother's age 19-24 years
Quantile

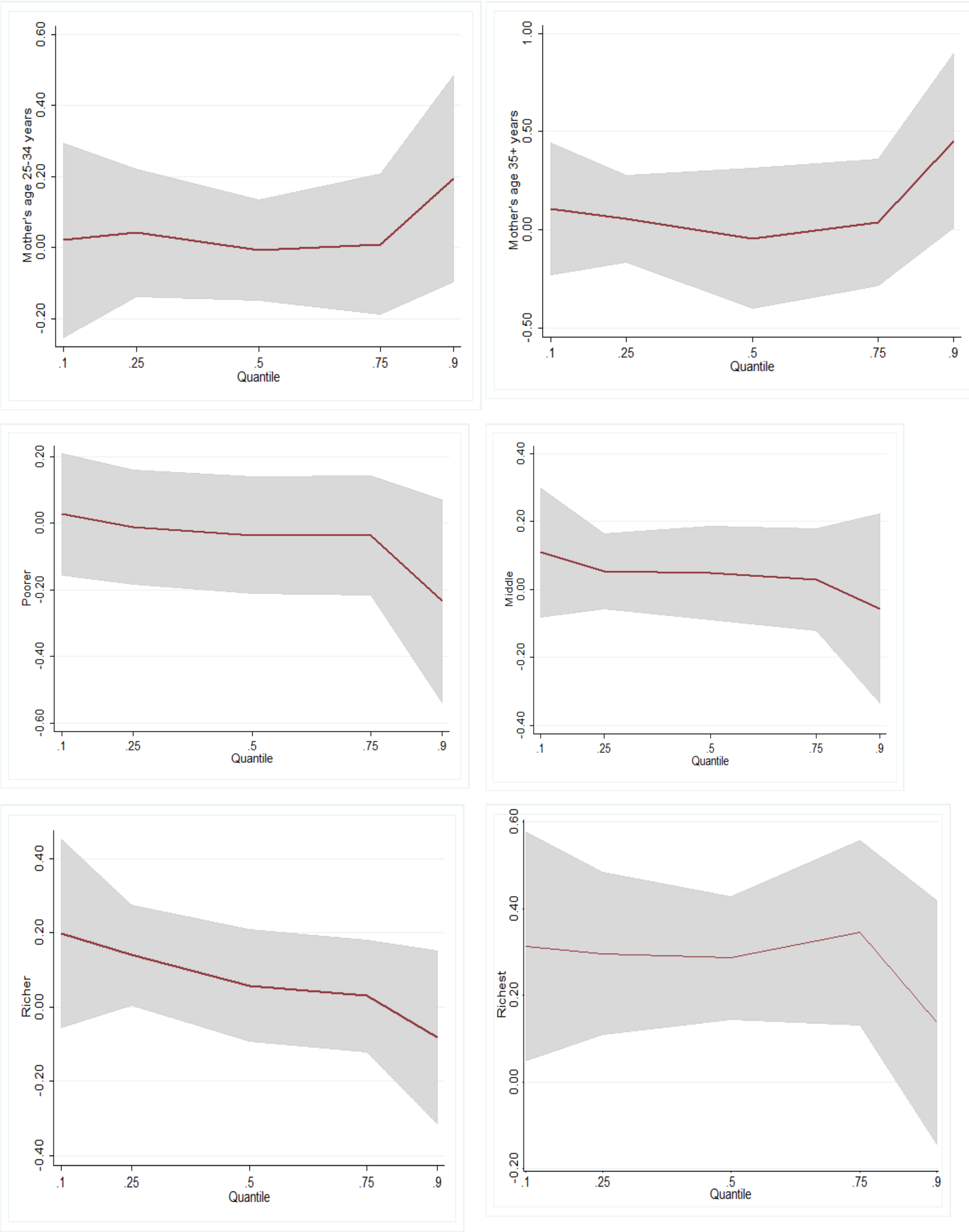

Figure 2: Estimates of quantile regression of three selected risk predictors (i.e. Child's age, Mother's age and Wealth index) for stunting (HAZ-score) in Bangladesh, 2017-18.

4. Discussion

The prevalence of stunting and related risk factors among Bangladeshi children under the age of five are described in this research. The Z scores for height for age was used to determine the extent of stunting among children under the age of five (HAZ). Findings demonstrated that the child's age has a significant adverse association with HAZ score. The relationship varied throughout the various segments of the conditional distribution of HAZ which is consistent with the findings of another study [62,66]. Results depict that children aged 24–59 months were at higher risk for stunting than children aged 0–23. This is consistent with findings from similar studies [24,67], and may be explained by the protective effect of breastfeeding. One of the main reason working behind this is the most of the children in Bangladesh are breastfed until 24 months and breastfeeding gradually declines with child age [2,68]. The higher risk of stunting at a later age may be due to the fact that children over the age of 24 months receive less parental attention and are actively playing, particularly in unsanitary conditions where they are susceptible to diseases such as enteric infections [26].

The level of parental education was found to be a major influence in stunting in our study. Our research found that mothers with a secondary or higher level of education had significantly fewer stunted children than uneducated mothers. A similar finding was discovered for paternal education. Other studies validated the findings [2,69]. Childhood vaccines, family planning, visits to local health clinics, and vitamin A supplementation are all influenced by parental education [70]. Furthermore, it is widely accepted that better education leads to higher earnings. As a result, higher family earnings allow parents to spend more on health care and good nutrition for their children, which may explain why educated parents have a lower stunting rate [71]. The findings of quantile regression demonstrated that birth order was negatively linked with HAZ-value. Moreover, religious status, and household's wealth index showed a positive correlation to the HAZ score in most of the cases. Data also reveal that birth order has a considerable adverse influence on the conditional distribution of HAZ-score of a child. Some of the findings of the current study having consistency with the outcome of another study [66] though they use BDHS-2014 survey data. This finding confirmed that children born at home have a higher risk of

malnutrition. The explanation for this could be a lack of sufficient care for children compared to the children who are born with health facilities. From health-care providers, parents received sufficient knowledge and guidance on child feeding practices and nutrition, which can be used as a measure to lessen the burden.

The household income is a contributing factor of child health status. Typically, wealthier families can ensure better medical care along with more nutritious food as well as can provide an improved and healthier living environment [72]. Children of middle-income, more affluent, and wealthiest families had a better level of HAZ scores as compared with the children come from the poorest families. Children from lower-income families were more likely to be stunted than children from higher-income families due to inadequate food consumption, a lack of basic health care, and a higher risk of infection. Furthermore, the quality of antenatal care is highly linked to one's wealth. This study corroborated findings from previous studies in other developing nations, indicating that household economic conditions are an important factor in the nutritional status of children in developing countries [2,73–75].

5. Conclusions

This paper has explored the influences of some selected child, maternal as well as household and health characteristics using the quantile regression method for determining child nutritional status at several points of the conditional distribution of the HAZ score. Among the predictors included in this study, the age, mother's BMI and mother's as well as father's educational status, number of antenatal care visits during pregnancy, household's wealth index were all found to be significant determinants of the Z score of height-for-age of a child. Household's wealth status was a key factor of stunting since it is directly associated with the affordability of food with high nutritional value, living in an improved and healthy environment and access to better health care services. In order to lessen the burden of malnutrition among children, the authors recommend that much more collaborative efforts by the government, NGOs and community organisations are necessary to improve the household income, especially for families in the rural area.

Along with the continuing programs for the betterment of child health status, the government of Bangladesh may need to design targeted nutritional intervention strategies with a clearer understanding of the focus group to lessen childhood stunting. Also, the easier entree to health information and inadequate health knowledge of parents, surveillance as well as assessment needs to be reviewed frequently with particular attention to vulnerable groups like children with poor maternal conditions such as mothers' with low BMI and educational status or who resided in the remote and regional areas. A healthy mother can deliver a healthy children, therefore, for enhancing the nutritional status of children, the attention of early intervention programs should be on children along with their mothers. Hence, the contributors of this paper would like to recommend that health as well as nutrition education also should be incorporated in the mass media programs and academic educational process in Bangladesh. Finally, the outcomes of this study would help practitioners and policy-makers to implement robust and cohesive programs to achieve the Sustainable Development Goals (SDGs) associated to child health in Bangladesh by 2030.

Supplementary Figure 1. Quantile regression estimates of selected risk predictors for childhood stunting (HAZ) in Bangladesh, 2017-18

Declarations

Consent to publication

Not applicable

Acknowledgements

None.

Financial Support

This research received no specific grant from any funding agency, commercial or not-for-profit sectors.

Competing of interests

None.

Ethics approval

It is not required as the survey was approved by the Ethics Committee of the ICF Macro at Calverton in the USA and by the Ethics Committee in Bangladesh.

Data availability

This study is based on the secondary dataset. One can access the data set via the following link *http://dhsprogram.com/data/available-datasets.cfm*.

Table 2: Quantile regression modelling results of selected risk predictors for stunting (HAZ score) for the under-5 children in Bangladesh, 2017-18.

Background characteristics	Q10 (95% CI)	Q25 (95% CI)	Q50 (95% CI)	Q75 (95% CI)	Q90 (95% CI)
Sex of child (ref=male)					
Female	-0.041 (-0.166, 0.083)**	-0.059 (-0.14, 0.023)*	-0.02 (-0.128, 0.088)	0.021 (-0.076, 0.119)**	-0.06 (-0.183, 0.064)**
Child's age (months) (ref<=6)					
7-12	-0.05 (-0.431, 0.330)	-0.167 (-0.385, 0.052)*	-0.124 (-0.338, 0.092)*	-0.202 (-0.464, 0.059)	-0.034 (-0.525, 0.456)
13-23	-0.579 (-0.977, -0.180)***	-0.627 (-0.857, -0.394)***	-0.592 (-0.806, -0.377)***	-0.583 (-0.873, -0.293)***	-0.526 (-0.991, -0.061)**
24- 35	-0.423 (-0.850, -0.003)**	-0.419 (-0.702, -0.137)***	-0.312 (-0.564, -0.059)***	-0.24 (-0.534, 0.054)*	-0.184 (-0.669, 0.301)
36-47	-0.188 (-0.692, 0.317)**	-0.263 (-0.612, 0.086)**	-0.166 (-0.409, 0.078)**	-0.067 (-0.324, 0.189)	0.062 (-0.516, 0.639)
48-59	0.188 (-0.366, 0.742)	0.263 (-0.035, 0.561)*	0.166 (-0.183, 0.514)	0.067 (-0.287, 0.421)	-0.062 (-0.565, 0.441)
Birth order (ref=1st order)					
2nd-3rd	0.008 (-0.168, 0.184)**	0.011 (-0.076, 0.098)**	0.05 (-0.044, 0.145)	0.032 (-0.097, 0.16)**	-0.046 (-0.247, 0.154)***
4th and higher	0.005 (-0.24, 0.249)	-0.09 (-0.217, 0.036)*	-0.028 (-0.198, 0.143)	0.069 (-0.173, 0.31)	-0.168 (-0.428, 0.092)**
Duration of breast-feeding (ref=never)					
<12 months	-0.728 (-1.747, 0.292)**	-0.873 (-1.946, 0.2)	-0.576 (-1.18, 0.028)*	-0.118 (-1.404, 1.167)*	-0.481 (-92.004, 91.042)
12 or more	-0.633 (-1.673, 0.408)**	-0.932 (-2.007, 0.142)*	-0.7 (-1.261, -0.14)**	-0.423 (-1.751, 0.905)***	-1.069 (-92.718, 90.58)*
Still breastfeeding	-0.699 (-1.692, 0.294)**	-0.792 (-1.817, 0.234)*	-0.539 (-1.103, 0.024)*	-0.292 (-1.611, 1.027)**	-1.253 (-92.835, 90.329)*
Religion (ref=muslim)					
Non-muslim	0.131 (-0.021, 0.282)*	-0.004 (-0.15, 0.141)	-0.039 (-0.185, 0.106)	0.061 (-0.171, 0.293)**	0.3 (0.002, 0.598)**

Background characteristics	Q10 (95% CI)	Q25 (95% CI)	Q50 (95% CI)	Q75 (95% CI)	Q90 (95% CI)
Mother's age (ref= Up to 18 years)					
19-24	0.004 (-0.17, 0.178)	0.007 (-0.124, 0.137)**	-0.032 (-0.194, 0.129)***	-0.005 (-0.161, 0.151)**	0.189 (-0.096, 0.474)*
25-34	0.02 (-0.232, 0.272)**	0.041 (-0.12, 0.202)*	-0.007 (-0.165, 0.15)*	0.009 (-0.178, 0.197)**	0.194 (-0.199, 0.586)**
35+	0.106 (-0.392, 0.605)***	0.056 (-0.126, 0.239)	-0.043 (-0.269, 0.182)***	0.038 (-0.216, 0.291)**	0.454 (-0.03, 0.937)*
Mother's Education level (ref= No Education)					
Primary	0.155 (-0.127, 0.437)	0.052 (-0.127, 0.23)	-0.057 (-0.335, 0.22)	-0.079 (-0.349, 0.19)	-0.063 (-0.441, 0.314)**
Secondary and above	0.294 (-0.033, 0.62)*	0.171 (-0.003, 0.345)**	-0.016 (-0.296, 0.264)**	0.047 (-0.225, 0.32)***	0.012 (-0.358, 0.381)*
Mother's BMI (ref= Underweight (<18.5))					
Normal (18.5-24.9)	0.158 (0.008, 0.307)**	0.181 (0.049, 0.312)**	0.185 (0.067, 0.303)**	0.276 (0.137, 0.415)***	0.202 (-0.074, 0.478)
Overweight (>=25)	0.162 (-0.059, 0.384)**	0.255 (0.104, 0.406)***	0.324 (0.176, 0.471)***	0.371 (0.233, 0.508)***	0.34 (0.066, 0.613)**
Father's Education level (ref= No Education)					
Primary	0.129 (-0.079, 0.338)	0.11 (-0.018, 0.237)*	0.066 (-0.082, 0.215)	0.161 (0.041, 0.281)**	0.113 (-0.226, 0.453)
Secondary and above	0.319 (0.138, 0.5)***	0.346 (0.24, 0.453)***	0.267 (0.129, 0.404)***	0.234 (0.073, 0.396)**	0.186 (-0.108, 0.48)*
Place of residence (ref=rural)					
Urban	-0.045 (-0.179, 0.089)	0.014 (-0.079, 0.106)*	0.093 (0, 0.186)**	0.039 (-0.053, 0.132)*	0.103 (-0.098, 0.304)
Place of delivery (ref= with health facility)					
Respondent's Home	-0.156 (-0.271, -0.04)**	-0.126 (-0.206, -0.045)**	-0.131 (-0.215, -0.046)**	-0.083 (-0.195, 0.028)**	-0.059 (-0.261, 0.143)***
Number of Antenatal care visit (ref=none)					
1-3	-0.025 (-0.229, 0.179)	-0.06 (-0.191, 0.072)	-0.019 (-0.147, 0.11)*	-0.063 (-0.261, 0.136)	-0.194 (-0.483, 0.096)**
4-6	-0.022 (-0.198, 0.154)**	-0.056 (-0.196, 0.084)**	0.031 (-0.108, 0.17)**	-0.024 (-0.254, 0.207)**	-0.087 (-0.373, 0.2)***

Background characteristics	Q10 (95% CI)	Q25 (95% CI)	Q50 (95% CI)	Q75 (95% CI)	Q90 (95% CI)
7+	0.068<br>(-0.323, 0.459)*	0.069<br>(-0.17, 0.308)***	0.083<br>(-0.099, 0.265)**	0.061<br>(-0.192, 0.314)***	-0.026<br>(-0.357, 0.306)*
Received BCG (ref=no)					
Yes	-0.384<br>(-0.587, -0.18)***	-0.436<br>(-0.598, -0.274)***	-0.427<br>(-0.606, -0.248)***	-0.535<br>(-0.797, -0.273)***	-99.177<br>(-99.461, -98.893)***
Received vitamin A (ref=no)					
Yes	-0.277<br>(-0.42, -0.133)***	-0.159<br>(-0.246, -0.072)***	-0.179<br>(-0.275, -0.083)***	-0.198<br>(-0.328, -0.069)**	-0.228<br>(-0.498, 0.042)*
Wealth index (ref= Poorest)					
Poorer	0.027<br>(-0.176, 0.23)	-0.012<br>(-0.151, 0.127)	-0.036<br>(-0.182, 0.111)*	-0.036<br>(-0.213, 0.142)*	-0.234<br>(-0.522, 0.054)**
Middle	0.109<br>(-0.082, 0.3)*	0.054<br>(-0.066, 0.174)***	0.049<br>(-0.105, 0.203)*	0.028<br>(-0.16, 0.217)**	-0.056<br>(-0.305, 0.194)***
Richer	0.198<br>(-0.048, 0.444)**	0.139<br>(0.003, 0.276)**	0.058<br>(-0.075, 0.191)***	0.03<br>(-0.118, 0.178)***	-0.082<br>(-0.426, 0.263)***
Richest	0.31<br>(0.06, 0.559)**	0.314<br>(0.142, 0.486)***	0.282<br>(0.099, 0.466)**	0.326<br>(0.13, 0.523)***	0.144<br>(-0.19, 0.477)**
Intercept	-1.519<br>(-2.715, -0.324)**	-0.61<br>(-1.644, 0.424)	-0.078<br>(-0.72, 0.565)	0.423<br>(-0.916, 1.762)	100.867<br>(9.13, 192.605)**

Notes: p-value<0.01***, 0.01<p-value<0.05**, 0.05<p-value<0.1*.

Reference

1. Victora CG, Adair L, Fall C, Hallal PC, Martorell R, Richter L, et al. Maternal and child undernutrition: consequences for adult health and human capital. Lancet. 2008;371: 340–357. doi:10.1016/S0140-6736(07)61692-4
2. Chowdhury TR, Chakrabarty S, Rakib M, Afrin S, Saltmarsh S, Winn S. Factors associated with stunting and wasting in children under 2 years in Bangladesh. Heliyon. 2020;6: e04849. doi:10.1016/j.heliyon.2020.e04849
3. Setianingsih, Permatasari D, Sawitri E, Ratnadilah D. Impact of Stunting on Development of Children Aged 12–60 Months. Adv Heal Sci Res. 2020;27: 186–189. doi:10.2991/ahsr.k.200723.047
4. National Institute of Population Research and Training (NIPORT), ICF. Bangladesh Demographic and Health Survey 2017-18. Dhaka, Bangladesh, and Rockville, Maryland, USA: NIPORT and ICF; 2020. Available: https://dhsprogram.com/pubs/pdf/FR344/FR344.pdf
5. Black RE, Victora CG, Walker SP, Bhutta ZA, Christian P, De Onis M, et al. Maternal and child undernutrition and overweight in low-income and middle-income countries. Lancet. 2013;382: 427–451. doi:10.1016/S0140-6736(13)60937-X
6. Eshete T, Kumera G, Bazezew Y, Marie T, Alemu S, Shiferaw K. The coexistence of maternal overweight or obesity and child stunting in low-income country: Further data analysis of the 2016 Ethiopia demographic health survey (EDHS). Sci African. 2020;9: e00524. doi:10.1016/j.sciaf.2020.e00524
7. UNICEF, WHO, World Bank. Levels and trends in child malnutrition: Key findings of the 2020 Edition of the Joint Child Malnutrition Estimates. Geneva WHO. 2020. doi:10.18356/6ef1e09a-en
8. World Health Organization. Global Nutrition Targets 2025: Policy Brief Series. World Heal Organ. 2014. doi:10.2165/00024677-200302060-00002
9. Development Initiatives. Global Nutrition Report 2017 - Nourishing the SDGs. 2017. Available: https://www.unscn.org/en/topics/sustainable-development-goals?idnews=1757
10. UNICEF. Stop Stunting in South Asia: A Common Narrative on Maternal and Child Nutrition | UNICEF South Asia. 2015. Available: https://www.unicef.org/rosa/reports/stop-stunting-south-asia-common-narrative-maternal-and-child-nutrition
11. Development Initiatives. 2020 Global Nutrition Report: Action on equity to end malnutrition. Glob Nutr Report's Indep Expert Gr. Bristol, UK; 2020. Available: https://globalnutritionreport.org/documents/566/2020_Global_Nutrition_Report_2hrssKo.pdf
12. Headey D, Heidkamp R, Osendarp S, Ruel M, Scott N, Black R, et al. Impacts of COVID-19 on childhood malnutrition and nutrition-related mortality. Lancet. 2020;396: 519–521.

doi:10.1016/S0140-6736(20)31647-0

13. Osendarp S, Akuoku J, Bank W, Black R, Headey D, Ruel M, et al. The potential impacts of the COVID-19 crisis on maternal and child undernutrition in low and middle income countries Results 4 Development Lawrence Haddad Global Alliance for Improved Nutrition. Res Sq. 2020. doi:10.21203/rs.3.rs-123716/v1

14. Fawzi MCS, Andrews KG, Fink G, Danaei G, McCoy DC, Sudfeld CR, et al. Lifetime economic impact of the burden of childhood stunting attributable to maternal psychosocial risk factors in 137 low/middle-income countries. BMJ Glob Heal. 2019;4: e001144. doi:10.1136/bmjgh-2018-001144

15. Gupta AK, Santhya KG. Proximal and contextual correlates of childhood stunting in India: A geo-spatial analysis. PLoS One. 2020;15: e0237661. doi:10.1371/journal.pone.0237661

16. Farah AM, Nour TY, Endris BS, Gebreyesus SH. Concurrence of stunting and overweight/obesity among children: Evidence from Ethiopia. Steinberg N, editor. PLoS One. 2021;16: e0245456. doi:10.1371/journal.pone.0245456

17. Hall J, Walton M, Ogtrop F Van, Guest D, Black K, Beardsley J. Factors influencing undernutrition among children under 5 years from cocoa-growing communities in Bougainville Handling editor Sanni Yaya. BMJ Glob Heal. 2020;5: e002478. doi:10.1136/bmjgh-2020-002478

18. Prüss A, Kay D, Fewtrell L, Bartram J. Estimating the burden of disease from water, sanitation, and hygiene at a global level. Environ Health Perspect. 2002;110: 537–542. doi:10.1289/ehp.110-1240845

19. Brown J, Cairncross S, Ensink JHJ. Water, sanitation, hygiene and enteric infections in children. Arch Dis Child. 2013;98: 629–634. doi:10.1136/archdischild-2011-301528

20. Cumming O, Cairncross S. Can water, sanitation and hygiene help eliminate stunting? Current evidence and policy implications. Matern Child Nutr. 2016;12: 91–105. doi:10.1111/mcn.12258

21. Vaivada T, Akseer N, Akseer S, Somaskandan A, Stefopulos M, Bhutta ZA. Stunting in childhood: An overview of global burden, trends, determinants, and drivers of decline. Am J Clin Nutr. 2020;112: 777S-791S. doi:10.1093/ajcn/nqaa159

22. Etzel RA. Reducing malnutrition: Time to consider potential links between stunting and mycotoxin exposure? Pediatrics. 2014. pp. 4–6. doi:10.1542/peds.2014-0827

23. Islam MS, Zafar Ullah AN, Mainali S, Imam MA, Hasan MI. Determinants of stunting during the first 1,000 days of life in Bangladesh: A review. Food Sci Nutr. 2020;8: 4685–4695. doi:10.1002/fsn3.1795

24. Adhikari RP, Shrestha ML, Acharya A, Upadhaya N. Determinants of stunting among children aged 0-59 months in Nepal: Findings from Nepal Demographic and health Survey, 2006, 2011, and 2016. BMC Nutr. 2019;5: 1–10. doi:10.1186/s40795-019-0300-0

25. Tydeman-Edwards R, Van Rooyen FC, Walsh CM. Obesity, undernutrition and the double burden of malnutrition in the urban and rural southern Free State, South Africa. Heliyon.

2018;4: e00983. doi:10.1016/j.heliyon.2018.e00983

26. Modern G, Sauli E, Mpolya E. Correlates of diarrhea and stunting among under-five children in Ruvuma, Tanzania; a hospital-based cross-sectional study. Sci African. 2020;8: e00430. doi:10.1016/j.sciaf.2020.e00430

27. Akram R, Sultana M, Ali N, Sheikh N, Sarker AR. Prevalence and Determinants of Stunting Among Preschool Children and Its Urban–Rural Disparities in Bangladesh. Food Nutr Bull. 2018;39: 521–535. doi:10.1177/0379572118794770

28. Manggala AK, Kenwa KWM, Kenwa MML, Sakti AAGDPJ, Sawitri AAS. Risk factors of stunting in children aged 24-59 months. Paediatr Indones. 2018;58: 205–212. doi:10.14238/pi58.5.2018.205-12

29. Ni’mah K, Nadhiroh SR. Factors related to stunting events in toddlers. Media Gizi Indones. 2015;10: 13–19. doi:10.20473/MGI.V10I1.13-19

30. Hailu BA, Bogale GG, Beyene J. Spatial heterogeneity and factors influencing stunting and severe stunting among under-5 children in Ethiopia: spatial and multilevel analysis. Sci Rep. 2020;10: 16427. doi:10.1038/s41598-020-73572-5

31. Budhathoki SS, Bhandari A, Gurung R, Gurung A, Kc A. Stunting Among Under 5-Year-Olds in Nepal: Trends and Risk Factors. Matern Child Health J. 2020;24: 39–47. doi:10.1007/s10995-019-02817-1

32. Li Z, Kim R, Vollmer S, Subramanian S V. Factors Associated With Child Stunting, Wasting, and Underweight in 35 Low- and Middle-Income Countries. JAMA Netw open. 2020;3: e203386. doi:10.1001/jamanetworkopen.2020.3386

33. Wali N, Agho KE, Renzaho AMN. Factors Associated with Stunting among Children under 5 Years in Five South Asian Countries (2014–2018): Analysis of Demographic Health Surveys. Nutrients. 2020;12: 3875. doi:10.3390/nu12123875

34. Sinha B, Taneja S, Chowdhury R, Mazumder S, Rongsen-Chandola T, Upadhyay RP, et al. Low-birthweight infants born to short-stature mothers are at additional risk of stunting and poor growth velocity: Evidence from secondary data analyses. Matern Child Nutr. 2018;14: e12504. doi:10.1111/mcn.12504

35. Indriani D, Lanti Y, Dewi R, Murti B, Qadrijati I. Prenatal Factors Associated with the Risk of Stunting: A Multilevel Analysis Evidence from Nganjuk, East Java. J Matern Child Heal. 2018;3: 294–300. doi:10.26911/thejmch.2018.03.04.07

36. Khan S, Zaheer S, Safdar NF. Determinants of stunting, underweight and wasting among children < 5 years of age: Evidence from 2012-2013 Pakistan demographic and health survey. BMC Public Health. BioMed Central Ltd.; 2019. p. 358. doi:10.1186/s12889-019-6688-2

37. Wicaksono F, Harsanti T. Determinants of stunted children in Indonesia: A multilevelanalysis at the individual, household, and community levels. Kesmas. 2020;15: 48–53. doi:10.21109/kesmas.v15i1.2771

38. Ekholuenetale M, Barrow A, Ekholuenetale CE, Tudeme G. Impact of stunting on early

childhood cognitive development in Benin: evidence from Demographic and Health Survey. Egypt Pediatr Assoc Gaz. 2020;68: 31. doi:10.1186/s43054-020-00043-x

39. Das S, Chanani S, Shah More N, Osrin D, Pantvaidya S, Jayaraman A. Determinants of stunting among children under 2 years in urban informal settlements in Mumbai, India: evidence from a household census. J Heal Popul Nutr. 2020;39: 10. doi:10.1186/s41043-020-00222-x

40. Astatkie A. Dynamics of stunting from childhood to youthhood in Ethiopia: Evidence from the Young Lives panel data. Assefa Woreta S, editor. PLoS One. 2020;15: e0229011. doi:10.1371/journal.pone.0229011

41. Agedew E, Chane T. Prevalence of Stunting among Children Aged 6–23 Months in Kemba Woreda, Southern Ethiopia: A Community Based Cross-Sectional Study. Adv Public Heal. 2015;2015: 1–6. doi:10.1155/2015/164670

42. Mazengia AL, Biks GA. Predictors of stunting among school-age children in Northwestern Ethiopia. J Nutr Metab. 2018;2018: 1–7. doi:10.1155/2018/7521751

43. Rachmi CN, Agho KE, Li M, Baur LA. Stunting, Underweight and Overweight in Children Aged 2.0–4.9 Years in Indonesia: Prevalence Trends and Associated Risk Factors. Zhang Y, editor. PLoS One. 2016;11: e015476. doi:10.1371/journal.pone.0154756

44. Mediani HS. Predictors of Stunting Among Children Under Five Year of Age in Indonesia: A Scoping Review. Glob J Health Sci. 2020;12: 83–95. doi:10.5539/gjhs.v12n8p83

45. Nshimyiryo A, Hedt-Gauthier B, Mutaganzwa C, Kirk CM, Beck K, Ndayisaba A, et al. Risk factors for stunting among children under five years: A cross-sectional population-based study in Rwanda using the 2015 Demographic and Health Survey. BMC Public Health. 2019;19: 175. doi:10.1186/s12889-019-6504-z

46. Rabaoarisoa CR, Rakotoarison R, Rakotonirainy NH, Mangahasimbola RT, Randrianarisoa AB, Jambou R, et al. The importance of public health, poverty reduction programs and women’s empowerment in the reduction of child stunting in rural areas of Moramanga and Morondava, Madagascar. Simeoni U, editor. PLoS One. 2017;12: e0186493. doi:10.1371/journal.pone.0186493

47. Boah M, Azupogo F, Amporfro DA, Abada LA. The epidemiology of undernutrition and its determinants in children under five years in Ghana. Zereyesus Y, editor. PLoS One. 2019;14: e0219665. doi:10.1371/journal.pone.0219665

48. Berhe K, Seid O, Gebremariam Y, Berhe A, Etsay N. Risk factors of stunting (chronic undernutrition) of children aged 6 to 24 months in Mekelle City, Tigray Region, North Ethiopia: An unmatched case-control study. Puebla I, editor. PLoS One. 2019;14: e0217736. doi:10.1371/journal.pone.0217736

49. Yaya S, Uthman OA, Kunnuji M, Navaneetham K, Akinyemi JO, Kananura RM, et al. Does economic growth reduce childhood stunting? A multicountry analysis of 89 Demographic and Health Surveys in sub-Saharan Africa. BMJ Glob Heal. 2020;5: e002042. doi:10.1136/bmjgh-2019-002042

50. Habimana S, Biracyaza E. Risk Factors Of Stunting Among Children Under 5 Years Of

Age In The Eastern And Western Provinces Of Rwanda: Analysis Of Rwanda Demographic And Health Survey 2014/2015. Pediatr Heal Med Ther. 2019;10: 115–130. doi:10.2147/phmt.s222198

51. Das S, Gulshan J. Different forms of malnutrition among under five children in Bangladesh: A cross sectional study on prevalence and determinants. BMC Nutr. 2017;3: 1. doi:10.1186/s40795-016-0122-2

52. Hasan MT, Soares Magalhaes RJ, Williams GM, Mamun AA. The role of maternal education in the 15-year trajectory of malnutrition in children under 5 years of age in Bangladesh. Matern Child Nutr. 2016;12: 929–939. doi:10.1111/mcn.12178

53. Kabir MR, Rahman M, Mamun MA Al, Islam H. Prevalence of malnutrition and associated factors affecting the nutritional status of Adivasi (tribal) children aged 24-59 months in Bangladesh. Asian J Med Biol Res. 2018;4: 178–185. doi:10.3329/ajmbr.v4i2.38253

54. Talukder A. Factors Associated with Malnutrition among Under-Five Children: Illustration using Bangladesh Demographic and Health Survey, 2014 Data. Children. 2017;4: 88. doi:10.3390/children4100088

55. Sultana P, Rahman MM, Akter J. Correlates of stunting among under-five children in Bangladesh: A multilevel approach. BMC Nutr. 2019;5. doi:10.1186/s40795-019-0304-9

56. Yeh CC, Wang KM, Suen YB. Quantile analyzing the dynamic linkage between inflation uncertainty and inflation. Probl Perspect Manag. 2009;7: 21–28.

57. Olsen CS, Clark AE, Thomas AM, Cook LJ. Comparing least-squares and quantile regression approaches to analyzing median hospital charges. Acad Emerg Med. 2012;19: 866–875. doi:10.1111/j.1553-2712.2012.01388.x

58. Hossain MM, Majumder AK. Determinants of the age of mother at first birth in Bangladesh: quantile regression approach. J Public Heal. 2019;27: 419–424. doi:10.1007/s10389-018-0977-6

59. Borooah VK. The height-for-age of Indian children. Econ Hum Biol. 2005;3: 45–65. doi:10.1016/j.ehb.2004.12.001

60. Aturupane H, Deolalikar AB, Gunewardena D. Determinants of Child Weight and Height in Sri Lanka: A Quantile Regression Approach. In: McGillivray M, Dutta I, Lawson D, editors. Health Inequality and Development. Studies in Development Economics and Policy, Palgrave Macmillan, London; 2011. doi:10.1057/9780230304673_4

61. Fenske N, Burns J, Hothorn T, Rehfuess EA. Understanding child stunting in India: A comprehensive analysis of socio-economic, nutritional and environmental determinants using additive quantile regression. PLoS One. 2013;8: e78692. doi:10.1371/journal.pone.0078692

62. Sharaf MF, Mansour EI, Rashad AS. Child nutritional status in Egypt: a comprehensive analysis of socio-economic determinants using a quantile regression approach. J Biosoc Sci. 2019;51: 1–17. doi:10.1017/S0021932017000633

63. Koenker R, Bassett G. Regression Quantiles. Econometrica. 1978;46: 33–50.

doi:10.2307/1913643

64. Yirga AA, Ayele DG, Melesse SF. Application of Quantile Regression: Modeling Body Mass Index in Ethiopia. Open Public Health J. 2018;11: 221–233. doi:10.2174/1874944501811010221

65. Lacalle-Calderon M, Perez-Trujillo M, Neira I. Fertility and Economic Development: Quantile Regression Evidence on the Inverse J-shaped Pattern. Eur J Popul. 2017;33: 1–31. doi:10.1007/s10680-016-9382-4

66. Rahman A, Hossain MM. Quantile regression approach to estimating prevalence and determinants of child malnutrition. J Public Heal. 2020; 1–17. doi:10.1007/s10389-020-01277-0

67. Ulak M, Chandyo RK, Mellander L, Shrestha PS, Strand TA. Infant feeding practices in Bhaktapur, Nepal: a cross-sectional, health facility based survey. Int Breastfeed J. 2012;7: 1–8. doi:10.1186/1746-4358-7-1

68. Dhungana GP. Nutritional Status and the Associated Factors in Under Five Years Children of Lamjung, Gorkha and Tanahun Districts of Nepal. Nepal J Stat. 2017;1: 15–18. doi:10.3126/NJS.V1I0.18814

69. Rahman A, Chowdhury S. Determinants of chronic malnutrition among preschool children in Bangladesh. J Biosoc Sci. 2007;39: 161–173. doi:10.1017/S0021932006001295

70. Semba RD, de Pee S, Sun K, Campbell AA, Bloem MW, Raju VK. Low intake of vitamin A-rich foods among children, aged 12-35 months, in India: Association with malnutrition, anemia, and missed child survival interventions. Nutrition. 2010;26: 958‑962. doi:10.1016/j.nut.2009.08.010

71. Chowdhury TR, Chakrabarty S, Rakib M, Saltmarsh S, Davis KA. Socio-economic risk factors for early childhood underweight in Bangladesh. Global Health. 2018;14: 1–12. doi:10.1186/s12992-018-0372-7

72. Rahman A, Chowdhury S. Determinants of chronic malnutrition among preschool children in Bangladesh. J Biosoc Sci. 2007;39: 161–173. doi:10.1017/S0021932006001295

73. Meshram II, Arlappa N, Balakrishna N, Laxmaiah A, Rao KM, Gal Reddy C, et al. Prevalence and determinants of undernutrition and its trends among pre-school tribal children of maharashtra state, India. J Trop Pediatr. 2012;58: 125–132. doi:10.1093/TROPEJ/FMR035

74. Hong R, Mishra V. Effect of wealth inequality on chronic under-nutrition in Cambodian children. J Heal Popul Nutr. 2006;24: 89–99. doi:10.3329/JHPN.V24I1.751

75. Kien VD, Lee H-Y, Nam Y-S, Oh J, Giang KB, Minh H Van. Trends in socioeconomic inequalities in child malnutrition in Vietnam: findings from the Multiple Indicator Cluster Surveys, 2000–2011. Glob Health Action. 2016;9: 29263. doi:10.3402/GHA.V9.29263

Click here to access/download
Supporting Information
Supplement Figure 1.docx

Authors responses to the review comments:

We would like to sincerely thank the Academic Editor for their valuable comments. We have considered all comments and then thoroughly revised and formatted the manuscript. A detailed response to each particular comment is provided in the tables as follows.

**Editorial Board Member comments:**	**Response**	**Note**
1. It has come to our attention that the manuscript seems to be a resubmission of the manuscript "PONE-D-21-04575", which was previously considered by the journal.<br>It is a requirement by the journal that any manuscript resubmitted after a previous rejection is accompanied by a rebuttal letter outlining the response to the original reviewers' comments, so that the editors can establish whether the original concerns have been addressed. Can you therefore please provide us with a point-by-point response to the comments provided by the original reviewers, outlining the revisions carried out since the original submission.	Thank you very much for your feedback. We add the author's response of the comments of the previous submission.	
2. It appears that you did not upload a Track Changes version of your manuscript file, which highlights any changes you made to your previous submission (PONE-D-21-04575).<br>Authors must submit Track Changes MS files as required by the journal on new submissions of previous PLOS ONE MSs.<br>At this time, please upload a Track Changes version of your manuscript to your File Inventory as an 'Other' file.	Thanks. We have submitted the track change versions along with this submission.	

**Editorial Board Member comments:**	**Response**	**Note**
ONE-D-21-04575<br>Assessing the Determinants of Stunting among under-5 Children in Bangladesh using Quantile Regression Approach<br>PLOS ONE<br><br>Thank you for submitting your manuscript for review by PLOS ONE. After careful consideration, we have decided that your manuscript does not meet our publication criteria and therefore must be rejected.<br>During our internal evaluation of the manuscript, we found significant text overlap between your submission and several previously published works, including:<br>https://link.springer.com/article/10.1007/s10389-020-01277-0<br>https://dhsprogram.com/pubs/pdf/FR311/FR311.pdf<br>www.cmamforum.org/Pool/Resources/Determinants-of-child-malnutrition-in-Bangladesh-2014-.pdf<br>We would like to make you aware that copying extracts from previous publications, especially outside the methods section, word-for-word is	Thanks. We appreciate your feedback. A careful revision has been conducted to minimize the similarities.<br><br>The required files along with the revised and formatted manuscript are uploaded in the journal system.	The revised text are highlighted in "red" color.

unacceptable. In addition, the reproduction of text from published reports has implications for the copyright that may apply to the publications.<br>In light of these concerns, we cannot consider your study for publication and are rejecting your manuscript.<br>I am sorry that we cannot be more positive on this occasion, but we hope that you appreciate the reasons for this decision.<br><br>Yours sincerely,<br>Susan Hepp<br>Associate Editor<br>PLOS ONE		

In conclusion, the revised version of the manuscript has been produced as per the review outcomes. So, we hope that you would be happy to see this greatly improved version. Once again, we would like to thank you all for your dedication, professional services and cooperation.
