# Supplementary material for: Prevalence and risk predictors of childhood stunting in Bangladesh
Source: PLoS One. 2023 Jan 26;18(1):e0279901. doi: 10.1371/journal.pone.0279901 (PMC9879476; doi:10.1371/journal.pone.0279901)
Supplement: Attachment — Submitted filename: Point-by-point response to reviewers.docx [file pone.0279901.s002.docx]

**Authors responses to the review comments:**

We would like to sincerely thank the Academic Editor for their valuable comments. We have considered all comments and then thoroughly revised and formatted the manuscript. A detailed response to each particular comment is provided in the tables as follows.

| **Editorial Board Member comments:** | **Response** | **Note** |
| --- | --- | --- |
| 1. It has come to our attention that the manuscript seems to be a resubmission of the manuscript “PONE-D-21-04575", which was previously considered by the journal.  It is a requirement by the journal that any manuscript resubmitted after a previous rejection is accompanied by a rebuttal letter outlining the response to the original reviewers' comments, so that the editors can establish whether the original concerns have been addressed. Can you therefore please provide us with a point-by-point response to the comments provided by the original reviewers, outlining the revisions carried out since the original submission. | Thank you very much for your feedback. We add the author’s response of the comments of the previous submission. |  |
| 2. It appears that you did not upload a Track Changes version of your manuscript file, which highlights any changes you made to your previous submission (PONE-D-21-04575).  Authors must submit Track Changes MS files as required by the journal on new submissions of previous PLOS ONE MSs.  At this time, please upload a Track Changes version of your manuscript to your File Inventory as an ‘Other’ file. | Thanks. We have submitted the track change versions along with this submission. |  |
| **Editorial Board Member comments:** | **Response** | **Note** |
| ONE-D-21-04575  Assessing the Determinants of Stunting among under-5 Children in Bangladesh using Quantile Regression Approach  PLOS ONE  Thank you for submitting your manuscript for review by PLOS ONE. After careful consideration, we have decided that your manuscript does not meet our publication criteria and therefore must be rejected.  During our internal evaluation of the manuscript, we found significant text overlap between your submission and several previously published works, including:  https://link.springer.com/article/10.1007/s10389-020-01277-0  https://dhsprogram.com/pubs/pdf/FR311/FR311.pdf  www.cmamforum.org/Pool/Resources/Determinants-of-child-malnutrition-in-Bangladesh-2014-.pdf  We would like to make you aware that copying extracts from previous publications, especially outside the methods section, word-for-word is unacceptable. In addition, the reproduction of text from published reports has implications for the copyright that may apply to the publications.  In light of these concerns, we cannot consider your study for publication and are rejecting your manuscript.  I am sorry that we cannot be more positive on this occasion, but we hope that you appreciate the reasons for this decision.  Yours sincerely,  Susan Hepp  Associate Editor  PLOS ONE | Thanks. We appreciate your feedback. A careful revision has been conducted to minimize the similarities.  The required files along with the revised and formatted manuscript are uploaded in the journal system. | The revised text are highlighted in “red” color. |

In conclusion, the revised version of the manuscript has been produced as per the review outcomes. So, we hope that you would be happy to see this greatly improved version. Once again, we would like to thank you all for your dedication, professional services and cooperation.
