# Supplementary material for: Prevalence and risk predictors of childhood stunting in Bangladesh
Source: PLoS One. 2023 Jan 26;18(1):e0279901. doi: 10.1371/journal.pone.0279901 (PMC9879476; doi:10.1371/journal.pone.0279901)
Supplement: Attachment — Submitted filename: Point-by-point response to reviewers.docx [file pone.0279901.s004.docx]

**Authors responses to the review comments:**

We would like to sincerely thank the Academic Editor for their valuable comments. We have considered all comments and then thoroughly revised and formatted the manuscript. A detailed response to each particular comment is provided in the tables as follows.

| **Editorial Board Member comments:** | **Response** | **Note** |
| --- | --- | --- |
| Please include the following items when submitting your revised manuscript:  A rebuttal letter that responds to each point raised by the academic editor and reviewer(s). You should upload this letter as a separate file labeled 'Response to Reviewers'.  A marked-up copy of your manuscript that highlights changes made to the original version. You should upload this as a separate file labeled 'Revised Manuscript with Track Changes'.  An unmarked version of your revised paper without tracked changes. You should upload this as a separate file labeled 'Manuscript'.  If you would like to make changes to your financial disclosure, please include your updated statement in your cover letter. Guidelines for resubmitting your figure files are available below the reviewer comments at the end of this letter. | Thanks. We appreciate your feedback. A careful revision has been conducted to minimize the similarities.  The required files along with the revised and formatted manuscript are uploaded in the journal system. | The revised text are highlighted in “red” color. |
| **Journal Requirements:** | **Response** | **Note** |
| When submitting your revision, we need you to address these additional requirements.  1. Please ensure that your manuscript meets PLOS ONE's style requirements, including those for file naming. The PLOS ONE style templates can be found at  <https://journals.plos.org/plosone/s/file?id=wjVg>  /PLOSOne_formatting_sample_main_body.pdf and  https://journals.plos.org/plosone/s/file?id=ba62  /PLOSOne_formatting_sample_title_authors_affiliations.pdf | Thank you very much for pointed out it. We have revised the manuscript following the PLOS ONE style. | The revised text are highlighted in “red” color.  Page: 1-18 |
| 2. Please provide additional details regarding participant consent. In the ethics statement in the Methods and online submission information, please ensure that you have specified (1) whether consent was informed and (2) what type you obtained (for instance, written or verbal, and if verbal, how it was documented and witnessed). If your study included minors, state whether you obtained consent from parents or guardians. If the need for consent was waived by the ethics committee, please include this information.  If you are reporting a retrospective study of medical records or archived samples, please ensure that you have discussed whether all data were fully anonymized before you accessed them and/or whether the IRB or ethics committee waived the requirement for informed consent. If patients provided informed written consent to have data from their medical records used in research, please include this information. | Thanks. It is not required as the survey was approved by the Ethics Committee of the ICF Macro at Calverton in the USA and by the Ethics Committee in Bangladesh. | The revised text are highlighted in “red” color.  Page: 7 |
| 3. Your ethics statement should only appear in the Methods section of your manuscript. If your ethics statement is written in any section besides the Methods, please move it to the Methods section and delete it from any other section. Please ensure that your ethics statement is included in your manuscript, as the ethics statement entered into the online submission form will not be published alongside your manuscript. | Thanks. We write it in the Methods section. | The revised text are highlighted in “red” color.  Page: 7 |
| 4. Please review your reference list to ensure that it is complete and correct. If you have cited papers that have been retracted, please include the rationale for doing so in the manuscript text, or remove these references and replace them with relevant current references. Any changes to the reference list should be mentioned in the rebuttal letter that accompanies your revised manuscript. If you need to cite a retracted article, indicate the article’s retracted status in the References list and also include a citation and full reference for the retraction notice. | Thank you very much. We carefully check the references in the both texts and reference list. |  |
| **Reviewer 1 comments:** | **Response** | **Note** |
| Dear Authors,  Thank you for the article. The topic is very relevant in the context of the increasing stunting rate in LMIC. However, the manuscript needs major revision, especially in the language aspect. The readers may get confused or lose interest. Kindly consider a professional language editing service before resubmission. | Thank you very much for your comments and feedback. We appreciate your feedback. A careful revision has been conducted to fix the grammatical issues. | The revised text are highlighted in “red” color.  Page: 1-18 |
| **Reviewer 2 comments:** | **Response** | **Note** |
| At first, I would like congratulate the authors on bringing out a manuscript on this important topic particularly in a n LMIC. However, it needs a few modifications as discussed below.  1. The manuscript needs a thorough language correction. | Thank you very much for your comments. We appreciate your feedback. A careful revision has been conducted to fix the grammatical issues. | The revised text are highlighted in “red” color.  Page: 1-18 |
| 2. Conclusion needs to be more specific and focused. | Thanks. The Conclusion section is revised as per your comments. | The revised text are highlighted in “red” color.  Page: 17-18 |
| 3. Discussion needs to be more elaborated with adequate reasoning.  Rest comments have been highlighted in the text | We appreciate your feedback. The Discussion section is revised as per your comments.  The manuscript is also revised as per all highlighted comments appeared in the text. | The revised text are highlighted in “red” color.  Page: 14-17 |

In conclusion, the revised version of the manuscript has been produced as per the review outcomes. So, we hope that you would be happy to see this greatly improved version. Once again, we would like to thank you all for your dedication, professional services and cooperation.
