# Supplementary material for: Prevalence and risk predictors of childhood stunting in Bangladesh
Source: PLoS One. 2023 Jan 26;18(1):e0279901. doi: 10.1371/journal.pone.0279901 (PMC9879476; doi:10.1371/journal.pone.0279901)
Supplement: Attachment — Submitted filename: Point-by-point response to reviewers.docx [file pone.0279901.s005.docx]

**Authors responses to the review comments:**

We would like to sincerely thank the Academic Editor for their valuable comments. We have considered all comments and then thoroughly revised and formatted the manuscript. A detailed response to each particular comment is provided in the tables as follows.

| **Academic Editor comments:** | **Response** | **Note** |
| --- | --- | --- |
| Please include the following items when submitting your revised manuscript:  A rebuttal letter that responds to each point raised by the academic editor and reviewer(s). You should upload this letter as a separate file labeled 'Response to Reviewers'.  A marked-up copy of your manuscript that highlights changes made to the original version. You should upload this as a separate file labeled 'Revised Manuscript with Track Changes'.  An unmarked version of your revised paper without tracked changes. You should upload this as a separate file labeled 'Manuscript'.  If you would like to make changes to your financial disclosure, please include your updated statement in your cover letter. Guidelines for resubmitting your figure files are available below the reviewer comments at the end of this letter. | Thanks. We appreciate your feedback. A careful revision has been conducted to minimize the similarities.  The required files along with the revised and formatted manuscript are uploaded in the journal system. | The revised text are highlighted in “red” color. |
| Please note that the previous reviewers have not been invited to assessed the revised manuscript yet. This is because the handling Academic Editor believes that additional copy editing is required for English language presentation before reviewers are Invited.  Please note one of the publication criteria at PLOS ONE (https://journals.plos.org/plosone/s/criteria-for-publication#loc-5 )is that articles must be presented in an intelligible fashion and written in clear, correct, and unambiguous English. PLOS ONE cannot provide copyediting for manuscripts, as such  we suggest you thoroughly copyedit your manuscript for language usage, spelling, and grammar. Further consideration will be dependent on this aspect of the manuscript. | Thank you very much. We revised the whole manuscript to fix the grammatical errors. | The revised text are highlighted in “red” color.  Page: 1-18 |
| **Journal Requirements:** | **Response** | **Note** |
| Please review your reference list to ensure that it is complete and correct. If you have cited papers that have been retracted, please include the rationale for doing so in the manuscript text, or remove these references and replace them with relevant current references. Any changes to the reference list should be mentioned in the rebuttal letter that accompanies your revised manuscript. If you need to cite a retracted article, indicate the article’s retracted status in the References list and also include a citation and full reference for the retraction notice. | Thank you very much for pointed out it. We check the reference list and we ensure that all are complete and correct. |  |
| While revising your submission, please upload your figure files to the Preflight Analysis and Conversion Engine (PACE) digital diagnostic tool, https://pacev2.apexcovantage.com/. PACE helps ensure that figures meet PLOS requirements. To use PACE, you must first register as a user. Registration is free. Then, login and navigate to the UPLOAD tab, where you will find detailed instructions on how to use the tool. If you encounter any issues or have any questions when using PACE, please email PLOS at figures@plos.org. Please note that Supporting Information files do not need this step. | Thanks. We already check all figures using PACE at the time of submit the revised version of the manuscript. |  |

In conclusion, the revised version of the manuscript has been produced as per the review outcomes. So, we hope that you would be happy to see this greatly improved version. Once again, we would like to thank you all for your dedication, professional services and cooperation.
